# Increased Th17 cells and IL-17A exist in patients with B cell acute lymphoblastic leukemia and promote proliferation and resistance to daunorubicin through activation of Akt signaling

**DOI:** 10.1186/s12967-016-0894-9

**Published:** 2016-05-12

**Authors:** Laixi Bi, Junqing Wu, Aifang Ye, Jianbo Wu, Kang Yu, Shenghui Zhang, Yixiang Han

**Affiliations:** Department of Hematology, The First Affiliated Hospital of Wenzhou Medical University, Wenzhou, 325015 China; Laboratory of Internal Medicine, the First Affiliated Hospital of Wenzhou Medical University, Wenzhou, 325015 China

**Keywords:** B-cell acute lymphoblastic leukemia, Th17 cells, IL-17A, Th1 cells

## Abstract

**Background:**

Immune regulation is crucial for the pathogenesis of B-cell acute lymphoblastic leukemia (B-ALL). It has been reported that Th17 cells as a newly identified subset of CD4^+^ T cells are involved in the pathogenesis of several hematological disorders. However, the role of Th17 cells in the pathophysiology of B-ALL is still unclear.

**Methods:**

The frequencies of T cells were determined by flow cytometry in the peripheral blood and bone marrow of 44 newly diagnosed B-ALL patients and 25 age-matched healthy donors. The cell viability and apoptosis were determined by CCK-8 assay and Annexin V staining, respectively. Western blot was applied to identify the level of Akt and Stat3 phosphorylation.

**Results:**

We assessed and observed a significantly increased frequency of Th17 cells and a drastically decreased frequency of Th1 cells in peripheral blood mononuclear cells and bone marrow mononuclear cells from newly diagnosed B-ALL patients compared with healthy donors. Furthermore, increased levels of Th17-related cytokines including IL-17, IL-21, IL-23, IL-1β, and IL-6 were presented in between blood and marrow in B-ALL patients. Both IL-17A and IL-21, two Th17-secreted cytokines, induced the proliferation of B-ALL cell line Nalm-6 and patient B-ALL cells isolated from B-ALL patients, herein either cytokine led to the phosphorylation of Akt and Stat3. Additionally, IL-17A promoted resistance to daunorubicin via activation of Akt signaling and the PI3K/Akt inhibitor LY294002 or perifosine almost completely rescued daunorubicin-induced cell death in B-ALL cells.

**Conclusions:**

Our findings suggest that elevated Th17 cells secrete IL-17A by which promotes the proliferation and resistance to daunorubicin in B-ALL cells through activation of Akt signaling. Th17 cells may represent a novel target to improve B-ALL immunotherapy.

**Electronic supplementary material:**

The online version of this article (doi:10.1186/s12967-016-0894-9) contains supplementary material, which is available to authorized users.

## Background

B-cell acute lymphoblastic leukemia (B-ALL) characterized by accumulation of immature lymphoid progenitors affects both children and adults. Great strides in development of effective treatments have led to a cure rate of more than 90 % in children, whereas the prognosis of adults remains poor [1]. Relapses, treatment resistance and serious infections are the leading causes of death in most cases. Pro-survival signals provided by tissue microenvironments, such as intricate crosstalk between B-ALL cells and CD4^+^ T cells, mesenchymal stromal cells, and various cytokines, contribute to maintain leukemic clones and promote resistance to chemotherapy in adult B-ALL patients [2].

Undoubtedly, immune dysfunction occurs in the bone marrow (BM) microenvironment of patients with B-ALL due to abnormal infiltrations of several immune cells. As one of the most important immune cells, CD4^+^ T cells are vital in the induction and control the differentiation of other immune cells in response to tumor-specific antigens and play a central role in initiating, regulating, and maintaining immune responses against cancer. Th1 cells produce interferon-γ (IFN-γ) and predominantly promote cell-mediated immunity, whereas Th2 cells secrete IL-4, IL-5 and IL-13. Th17 cells, named for their signature production of IL-17A, also secrete IL-17F, IL-21, and IL-22, thereby inducing an enormous tissue reaction due to the broad distribution of the IL-17 and IL-21 receptors and exerting a crucial role in the development of inflammatory and autoimmune diseases [3–6]. In addition, Th17 cells are implicated in many cancers, but their role in cancer has not been well elucidated and remains under debate [7–10]. It is now well accepted that Th1 cells and their secreted signature cytokine IFN-γ exhibit robust anti-tumor activity [11, 12]. Several studies have shown that IFN-γ inhibits Th17 cell differentiation and IL-17 inhibits Th1 cell differentiation and IFN-γ production [7, 13–15], suggesting that Th1 and Th17 cells appear to have a reciprocal relation in function. Therefore, it makes sense to investigate the interplay of Th1 and Th17 cells in the tumor microenvironment. B-ALL represents an ideal model to assess the crosstalk between tumor and the immune system as the disease is usually widely disseminated, so that immune cells in the peripheral blood (PB) and BM are in close contact with B-ALL cells. The immune system fails to identify and eliminate B-ALL cells, by which contributes to the development of B-ALL.

In the present study, we demonstrate that elevated frequencies of Th17 cells and Th17-related cytokines and reduced frequencies of Th1 cells are presented in PB and BM from B-ALL patients; Th17-secreted cytokines IL-17A and IL-21 activate the Akt signaling and Stat3 signaling, and subsequently stimulate the proliferation in B-ALL cell line Nalm-6 and primary B-ALL cells; IL-17A also promotes resistance to daunorubicin through activation of Akt signaling.

## Methods

### Patients and samples

A total of 44 patients with newly diagnosed B-ALL (age range 15–68 years, median age 33 years) were enrolled in this study from July 2009 and February 2015 in our center. All patients with B-ALL were diagnosed according to the World Health Organization classification system [16]. Twenty-five age-matched healthy donors for control were simultaneously enrolled in this study. This study was approved by the Institutional Review board of the First Affiliated Hospital of Wenzhou Medical University and informed consent was obtained from all participants in accordance with the Declaration of Helsinki protocol.

### Flow cytometric analysis

The frequencies of Th17 cells and Th1 cells were analyzed as previously described [7]. Briefly, peripheral blood mononuclear cells (PBMCs) and bone marrow mononuclear cells (BMMCs) from B-ALL patients and healthy donors were stimulated with 40 ng/ml phorbol 12-myristate13-acetate (PMA) and 1 μg/ml ionomycin for 5 h in the presence of 1 μg/ml brefeldin A (BFA; all from Sigma-Aldrich, St.louis, MO, USA). The cells were subsequently surface stained with a combination of PerCP-conjugated anti-CD3 and FITC-conjugated anti-CD8, fixed and permeabilized and intracellularly stained with PE-conjugated anti-IL-17A and APC-conjugated anti-IFN-γ (all from BD Biosciences, San Jose, CA, USA). Stained cells were acquired and analyzed using CellQuest software on a FACSCalibur instrument (BD Biosciences).

### ELISA for cytokine measurements

The plasma samples were obtained from PB and BM after centrifugation and stored at −80 °C until used. Human IL-17 and IL-21 were assayed using ELISA kits purchased from BioLegend (San Diego, CA, USA) with the assay sensitivities of 2.0 and 16.0 pg/ml, respectively. Human IL-23, IL-1β, and IL-6 were measured using ELISA kits purchased from R&D systems (Minneapolis, MN, USA) with the assay sensitivities of 15.0, 1.0, and 0.7 pg/ml, respectively.

### Quantitative RT-PCR for gene expression analysis

Total RNA was extracted from mononuclear cells using TRIzol reagent (Invitrogen, Carlsbad, CA, USA) and was subsequently reverse transcribed into cDNA according to the manufacturer’s guidelines. Then, quantitative PCR was performed using SYBR Green PCR master mix (TaKaRa, Dalian, China) with respective primer pairs (for primers sequences, see Additional file 1: Table S1) in triplicate on an ABI 7500 Real-Time instrument. Expression data were normalized to GAPDH as an endogenous control and the relative expression levels were evaluated using the 2^−ΔΔCt^ method.

### Cell Counting Kit-8 (CCK-8) assay and apoptosis assay

CD19^+^ cells were isolated from PBMCs or BMMCs of B-ALL patients (thereafter as patient B-ALL cells) using B Cell Isolation Kit II (Miltenyi Biotec, Bergisch Gladbach, Germany) according to the manufacture’s protocols. B-ALL cell line Nalm-6 was a gift from Dr. Chiqi Chen (School of Medicine, Jiaotong University, Shanghai, China). After Nalm-6 cells and patient B-ALL cells were treated or untreated with IL-17, IL-21 (both from Peprotech, Rocky Hill, NJ, USA), daunorubicin, LY294002 or perifosine (Selleck Chemicals, Houston, TX, USA), the cell viability and apoptosis were measured. The cell viability was determined using CCK-8 assay (Dojindo, Kumamoto, Japan) according to the manufacturer’s instruction. The absorbance was read at 450 nm using an ELISA reader (ELx800; Bio-Tek Instruments, Winooski, VT, USA). The apoptosis was determined using the Annexin V binding assay as previously described [17].

### Western blot assays

Western blot analysis was performed as previously described [18, 19]. Briefly, after treatment with IL-17A or IL-21, Nalm-6 cells and patient B-ALL cells were collected, lysed, and further subjected to western blot with antibodies specific for phospho-Akt (Ser473), Akt, GAPDH (Cell Signaling Technology, Beverly, MA, USA), phospho-Stat3 (Tyr705), or Stat3 (Bioworld Technology, St.Louis Park, MN, USA), respectively.

### Induction assay

CD4^+^ T cells were isolated from PBMCs of B-ALL patients using CD4^+^ T cell isolation kit (Miltenyi Biotec) according to the manufacturer’s instructions. CD4^+^ T cells (2 × 10^5^) were cultured in 1 ml of complete medium in 48-well plates stimulated with plate-bound anti-CD3 antibody (OKT3, 4 μg/ml; eBioscience, San Diego, CA, USA) and IL-2 (300 units/ml) with or without Nalm-6 cells. After 14 days, Th17 cells were determined after stimulation with PMA and ionomycin in the presence of BFA.

### Statistical analysis

Data are presented as mean ± SEM, and statistical analyses were performed by a one-way analysis of variance or Mann–Whitney test. *P* values less than 0.05 were considered statistically significant.

## Results

### Increased Th17 cells and decreased Th1 cells in B-ALL patients

**Fig. 1:**
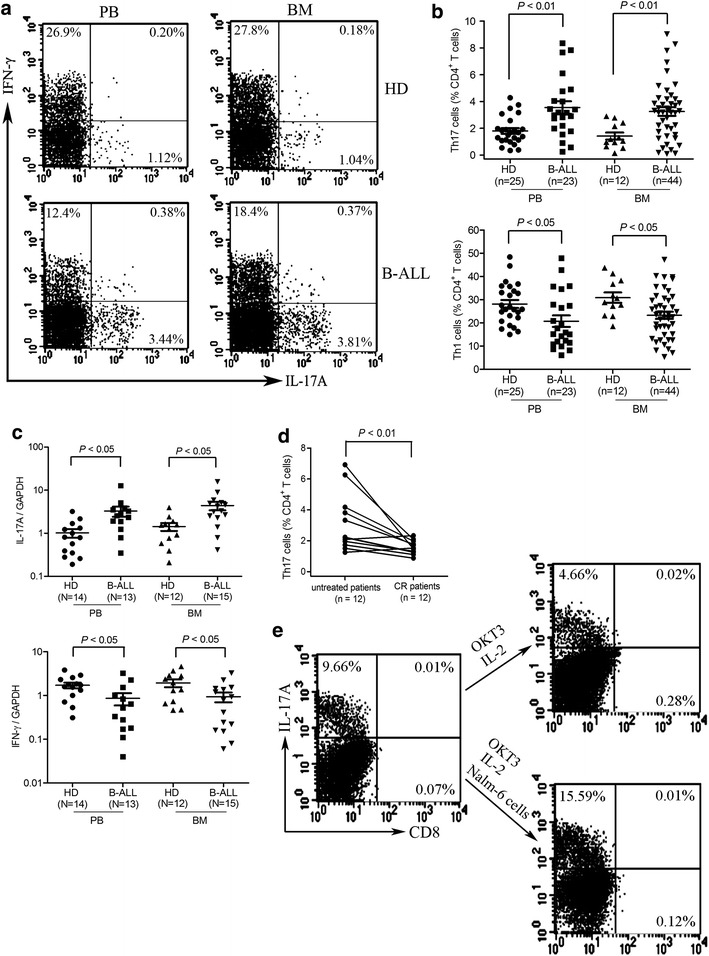
Th17 cells increase with reduced Th1 cells in freshly isolated PBMCs and BMMCs from patients with B-ALL. **a** PBMCs and BMMCs were separated and stimulated with PMA and ionomycin for 5 h in the presence of brefeldin A and subsequently stained with antibodies against CD3, CD8, intracellular IL-17A or IFN-γ. Flow cytometric analysis was used to determine the Th17 and Th1 cell frequencies. Representative *dot plots* using matching peripheral blood and bone marrow samples from B-ALL patients and healthy donors (HD) were shown. **b** Statistical data for frequencies of Th17 and Th1 cells within CD4^+^ T population were shown. **c** Total RNA was extracted from CD4^+^ T cells isolated from B-ALL patients and HDs and reverse transcribed into cDNA and subsequently determined for IL-17A and IFN-γ mRNA expression using quantitative PCR. **d** The frequencies of Th17 cells were significantly decreased in BM when B-ALL patients achieved complete remission (CR). **e** CD4^+^ T cells were cultured with or without Nalm-6 cells for 14 days in the presence of OKT3 plus IL-2 (300 units/ml). Then, frequencies of Th17 cells were determined after stimulation with PMA plus ionomycin

Th17 cells have been reported to be enriched in hematological malignancies including acute myeloid leukemia, multiple myeloma, and T-cell acute lymphoblastic leukemia [7, 15, 20, 21]. To investigate whether Th17 cells are also enriched in B-ALL, we evaluated the frequency of Th17 cells based on cytokine patterns after in vitro stimulation with PMA plus ionomycin in short-term culture. As shown in Fig. 1a, b, the frequencies of Th17 cells were 3.5 ± 0.46 % in B-ALL PBMCs compared with 1.8 ± 0.21 % in healthy donor PBMCs (*P* < 0.01), and 3.2 ± 0.32 % in B-ALL BMMCs compared with 1.4 ± 0.26 % in healthy donor BMMCs (*P* < 0.01), suggesting that Th17 cells are highly enriched in both PB and BM from B-ALL patients. We simultaneously analyzed the frequency of Th1 cells and found that the frequency of Th1 cells was significantly decreased in both PBMCs and BMMCs from B-ALL patients compared with those from healthy donors (Fig. 1a and b). Quantitative RT-PCR was used to measure the mRNA levels of IL-17A and IFN-γ in both PBMCs and BMMCs and found that increased expression of IL-17A and decreased expression of IFN-γ were presented in both PBMCs and BMMCs from B-ALL patients compared with those from healthy donors (Fig. 1c). Taken together, these findings suggest that Th17 cells are increased and Th1 cells are decreased in both PB and BM from B-ALL patients. Furthermore, when B-ALL patients achieved complete remission, the frequencies of Th17 cells were markedly decreased in BM compared with those from previously untreated patients (Fig. 1d).

Because increased Th17 cells were presented in B-ALL patients, we next investigated whether B-ALL cells drive the expansion of Th17 cells. We cultured bulk CD4^+^ T cells from B-ALL patients in the presence of IL-2 in OKT3-coated plates with or without Nalm-6 cells. As shown in Fig. 1e, the percentage of Th17 cells increased in CD4^+^ T cells cultured with Nalm-6 cells in the presence of OKT3 plus IL-2, whereas the percentage of Th17 cells decreased in CD4^+^ T cells cultured with OKT3 plus IL-2. These data indicate that the expansion of Th17 cells may be attributed to the interplay with B-ALL cells.

### Th17 cell-related cytokines in B-ALL patients

**Fig. 2:**
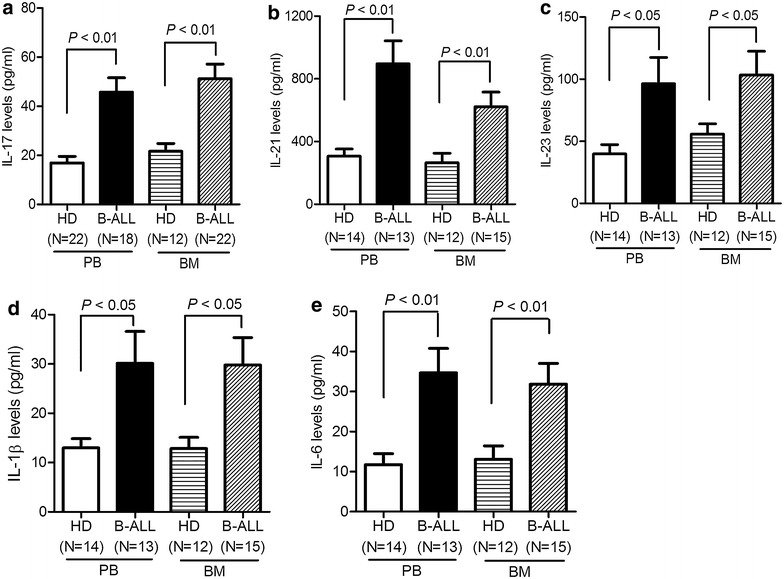
The levels of Th17-associated cytokines were increased in PB and BM samples from patients with B-ALL. The PB and BM samples were aspirated from B-ALL patients and healthy donors (HD) and determined for the levels of IL-17 (**a**), IL-21 (**b**), IL-23 (**c**), IL-1β (**d**), and IL-6 (**e**) using ELISA. Statistical data were expressed as mean ± SEM

IL-17A is the signature cytokine secreted by Th17 cells and contributes to Th17-mediated diseases. IL-21 is produced by Th17 cells and promotes or sustains Th17 lineage commitment [22]. IL-23, IL-1β, and IL-6 regulate the establishment and clonal expansion of Th17 cells. To further confirm elevated existence of Th17 cells in B-ALL patients, we measured the levels of Th17-related cytokines. We observed significant increases in levels of plasma IL-17A and IL-21 in PB and BM from newly diagnosed B-ALL patients compared with those from healthy donors (Fig. 2a and b). Higher levels of IL-23, IL-1β, and IL-6 were also observed in PB and BM from B-ALL patients compared with those from healthy donors (Fig. 2c–e). Taken together, these findings suggest that elevated Th17 cells appear to exist in the PB and BM microenvironment in B-ALL patients.

### Two Th17-related cytokines, IL-17A and IL-21, promote the proliferation of B-ALL cells via activation of the Akt and Stat3 signaling

**Fig. 3:**
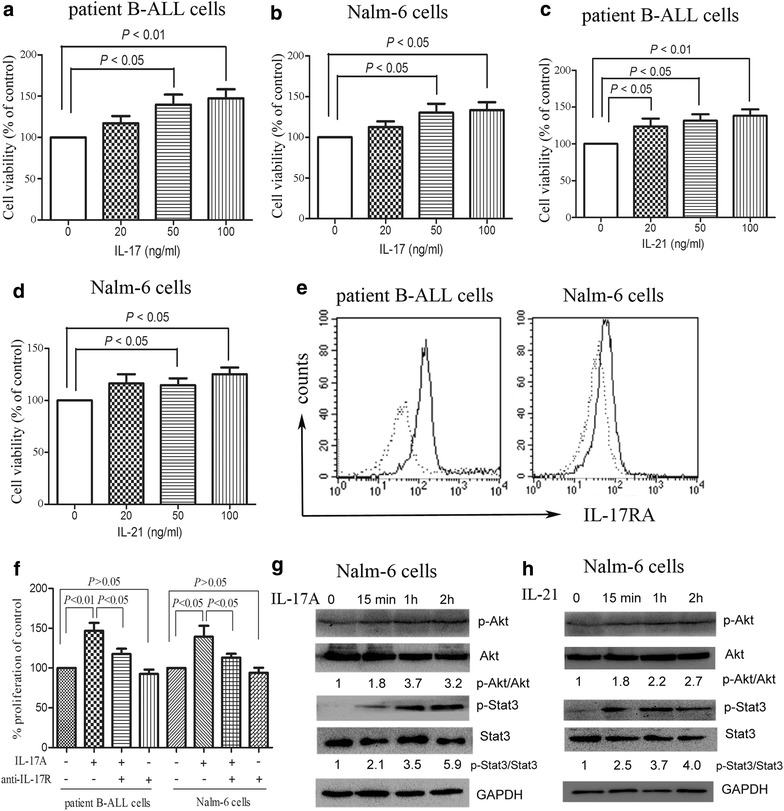
IL-17A promotes the proliferation of B-ALL cells via activation of IL-17R signaling. **a**–**d** Patient B-ALL cells isolated from 4 B-ALL patients and B-ALL cell line Nalm-6 were incubated in the presence or absence of IL-17A (50 ng/ml) or IL-21 (50 ng/ml) for 7 days and then, CCK-8 assay was used to assess cell viability. Statistical data representing at least three independent experiments are presented as the viability in the presence of IL-17A or IL-21 compared with control. **e** Representative histograms were presented for the IL-17RA expression of patient B-ALL cells and Nalm-6 cells, determined using PE-conjugated anti-CD217 antibody (*solid line*) or mouse IgG1 antibody (*dotted line*) by flow cytometry. **f** Patient B-ALL cells and Nalm-6 cells were incubated with or without IL-17A (50 ng/ml) in the presence or absence of anti-IL-17R antibody (3 μg/ml) for 7 days and then, the cell viability was determined using CCK-8 assay. **g**, **h** Western blot analysis showed the phosphorylation of Akt and Stat3 were prominently increased within 2 h in Nalm-6 cells stimulated with IL-17A (50 ng/ml) or IL-21(50 ng/ml). Images representing four independent experiments were shown

As Th17 cells secrete a variety of cytokines, including IL-17A, IL-17F, IL-21, and IL-22, their effects on B-ALL cells could be attributed to the actions of any one or a combination of these cytokines. We have demonstrated that B-ALL cell line Nalm-6 promotes the differentiation and expansion of Th17 cells (Fig. 1e), which evokes our interest to investigate whether Th17 cells have a mutual effect on B-ALL cells. As shown in Fig. 3a and b, IL-17A potently promoted the proliferation of patient B-ALL cells and Nalm-6 cells. IL-21, another main cytokine secreted by Th17 cells, also promoted the proliferation of patient B-ALL cells and Nalm-6 cells (Fig. 3c and d). It has been reported that interleukins exert their effects via specific cell surface receptors. Therefore, we measured the expression of IL-17 receptor A (IL-17RA) and found that the IL-17RA was weakly expressed on patient B-ALL cells and Nalm-6 cells (Fig. 3e). We used anti-IL-17R antibody to further determine the effect of IL-17RA signaling in IL-17-induced B-ALL cell proliferation. As shown in Fig. 3f, although anti-IL-17R antibody alone did not significantly affect cell viability, it significantly inhibited the proliferation of B-ALL cells in the presence of IL-17A.

Both the PI3K/Akt and Jak2/Stat3 signaling have been shown to play pivotal roles in tumor cell proliferation, survival, invasion and immunosuppression in many tumors including leukemia [23, 24]. Therefore, we analyzed these two signalings in B-ALL cells stimulated with IL-17A or IL-21. As shown in Fig. 3g and h, IL-17A or IL-21 stimulation promoted the phosphorylation of Akt and Stat3 in Nalm-6 cells. These findings indicate that Th17 cells exhibit a pro-tumor effect on B-ALL cells through secreting IL-17 and IL-21.

### IL-17A promotes resistance to daunorubicin via activation of the Akt signaling

**Fig. 4:**
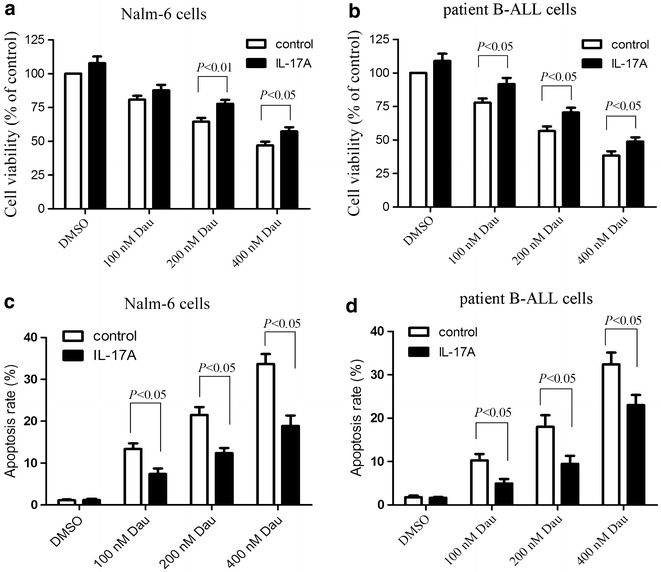
IL-17A promotes resistance to daunorubicin. Nalm-6 cells and patient B-ALL cells isolated from 4 B-ALL patients were cultured for 24 h in complete medium with or without IL-17A at 50 ng/ml and subsequently supplemented with daunorubicin at 100, 200, or 400 nM as indicated for 24 h. The cell viability was determined using CCK-8 assay, and apoptosis was determined using Annexin V staining. IL-17A promoted the proliferation and reduced apoptosis of Nalm-6 cells (**a**, **c**) and patient B-ALL cells (**b**, **d**). Statistical data representing at least 3 independent experiments were shown

**Fig. 5:**
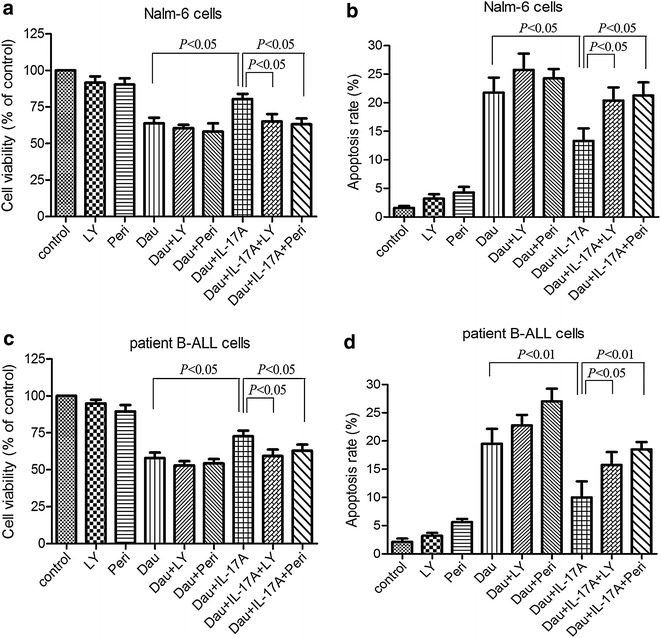
IL-17A-induced resistance to daunorubicin is dependent on Akt activation. Nalm-6 cells (**a**, **b**) and patient B-ALL cells (**c**, **d**) isolated from 4 B-ALL patients were pre-treated with the PI3K/Akt inhibitor LY294002 at 10 μM or perifosine at 2 μM for 2 h and treated with or without IL-17A at 50 ng/ml for 24 h. Then, the cells were further treated with daunorubicin at 200 nM for 24 h. The cell viability and apoptosis were determined using CCK-8 assay and Annexin V staining, respectively. Statistical data representing at least three independent experiments were shown

We had demonstrated above that IL-17A promoted the proliferation of B-ALL cells through activation of the Akt signaling (Fig. 3). As the PI3K/Akt signaling is implicated in resistance to anthracycline-based therapies [25], we tested whether IL-17A mediates resistance to daunorubicin, a chemotherapeutic drug for the treatment of B-ALL. Thus, Nalm-6 cells and patient B-ALL cells were pre-incubated with IL-17A and subsequently treated with daunorubicin. As shown in Fig. 4a and b, IL-17A treatment attenuated daunorubicin-induced cytotoxicities in Nalm-6 cells and patient B-ALL cells. Specifically, IL-17A also suppressed daunorubicin-induced apoptosis (Fig. 4c, d; Additional file 2: Figure S1, Additional file 3: Figure S2). To delineate the role of PI3K/Akt signaling in IL-17A-mediated resistance to daunorubicin, we treated these cells with the PI3K/Akt inhibitor LY294002 or perifosine. LY294002 or perifosine could abrogate IL-17A-mediated protection from daunorubicin-induced cell death in Nalm-6 cells whereas these two PI3K/Akt inhibitors alone almost did not affect cytotoxicity (Fig. 5a, b). LY294002 or perifosine also abrogated resistance to daunorubicin in patient B-ALL cells induced by IL-17A (Fig. 5c, d). Taken together, these data suggest that IL-17A protects from daunorubicin-induced cell death through activation of the Akt signaling.

## Discussion

Th17 cells contribute to protection against fungal and parasitic infections and participate in various inflammatory and autoimmune diseases. Furthermore, Targeted IL-17 depletion has already been accepted as a therapeutic strategy and is now being tested in clinical trials for several human autoimmune diseases [26, 27]. Whether Th17 cells and IL-17 also play similar roles in tumor pathogenesis and progression deserves further study. Here we demonstrate that increased Th17 cells and decreased Th1 cells are presented in PB and BM from B-ALL patients. IL-17A or IL-21, two cytokines mainly produced by Th17 cells, can promote the proliferation of B-ALL cells.

The interplay between immune cells and tumor cells contributes importantly in the induction of immune tolerance and tumor progression at tumor sites. Elevated proportions of Th17 cells have been identified in the total CD4^+^ T cell populations in several different human solid tumors and hematological malignancies including ovarian, prostate carcinomas, multiple myeloma, and acute myeloid leukemia [7, 20, 28, 29]. Abousamra NK et al. [30] have reported that Th17 cells were increased in PB in newly diagnosed ALL patients and a significantly longer overall survival in patients with high Th17 levels. Similarly, the present study also demonstrated that Th17 cells were increased in both PB and BM in newly diagnosed patients with B-ALL, a main subtype of ALL [1], indicating that B-ALL cells secrete critical cytokines required for the differentiation and expansion of Th17 cells. Some cytokines, such as IL-1β, IL-6, and IL-23, have been shown to promote the generation and differentiation of human Th17 cells [7]. Higher levels of IL-23, IL-1β, and IL-6 were observed in B-ALL patients, suggesting that the milieu contributes to the expansion of Th17 cells.

Malignant B cells isolated from B-cell NHL patients have been shown to induce the differentiation of regulatory T cells in vitro [31]. In the present study, B-ALL cell line Nalm-6 also promoted the differentiation and expansion of Th17 cells from CD4^+^ T cells, similar to ovarian tumor cells [28]. Undoubtedly, Th17 cells also secrete cytokines to affect B-ALL cells. It has been well identified that IL-17A plays an important role in promoting cancer growth. The PI3K/Akt and Jak2/Stat3 signaling are two important signalings implicated in IL-17A-mediated proliferation. In the present study, IL-17A stimulation promoted the proliferation of B-ALL cells through activation of Akt and Stat3 signaling. Obviously, blockade of IL-17RA signaling almost completely abrogated IL-17A-induced proliferation in B-ALL cells.

In healthy B cells, IL-21 induces differentiation of naïve and memory B cells into antibody-producing plasma cells [32]. However, IL-21 exerts diverse effects based on the histology of malignant B cells. For example, IL-21 stimulation induces apoptosis of chronic lymphocytic leukemia and diffuse large B-cell lymphoma cells while promoting the growth of myeloma and waldenstrom macroglobulinemia tumor cells [33–36]. In the present study, we focused on the role of IL-21 in B-ALL cells, to our knowledge which has not been discussed before. As expected, IL-21 promoted the proliferation of Nalm-6 cells and patient B-ALL cells, suggesting that it should work together with IL-17A to facilitate the expansion of B-ALL cells. Furthermore, the addition of IL-21 resulted in robust phosphorylation of Akt and Stat3 at 15 min post-stimulation, implying that the PI3K/Akt and Jak2/Stat3 signaling have been activated quickly, and these two signalings contribute to the proliferation effect.

Daunorubicin as a chemotherapeutic agent has been used clinically to treat B-ALL patients for several decades [37]. Several factors, such as the overexpression of microRNA-125b, influence resistance to daunorubicin [38], and whether IL-17A is an important factor remains unclear. In the present study, IL-17A resulted in resistance to daunorubicin against B-ALL cells, in which the Akt signaling play a key role, because a PI3K/Akt inhibitor LY294002 or perifosine almost blocks the protective effects of IL-17A.

## Conclusions

We found that increased Th17 cells and decreased Th1 cells were presented in PB and BM from patients with B-ALL. IL-17A or IL-21 could promote the proliferation of B-ALL cells through activation of Akt and Stat3 signaling. The PI3K/Akt inhibitor LY294002 or perifosine almost completely abrogated IL-17A-mediated protection from daunorubicin-induced cell death in B-ALL cells. Therefore all of the above data suggested that Th17 cells might serve as a potential target for B-ALL immunotherapy.

## Additional files

10.1186/s12967-016-0894-9 The sequences of the primers used for real-time qPCR.
10.1186/s12967-016-0894-9 IL-17A reduces apoptosis to daunorubicin in Nalm-6 cells. Nalm-6 cells were cultured for 24 h in complete medium with or without IL-17A at 50 ng/ml and subsequently supplemented with daunorubicin at 100, 200, or 400 nM as indicated for 24 h. Apoptosis was determined using Annexin V staining on a flow cytometry. Images representing 3 independent experiments were shown.
10.1186/s12967-016-0894-9 IL-17A reduces apoptosis to daunorubicin in patient B-ALL cells. Patient B-ALL cells isolated from 4 patients with B-ALL were cultured for 24 h in complete medium with or without IL-17A at 50 ng/ml and subsequently treated with daunorubicin at 100, 200, or 400 nM as indicated for 24 h. Apoptosis was measured using Annexin V staining on a flow cytometry. Images representing at least 3 independent experiments were shown.
