# Supplementary material for: Increased Th17 cells and IL-17A exist in patients with B cell acute lymphoblastic leukemia and promote proliferation and resistance to daunorubicin through activation of Akt signaling
Source: J Transl Med. 2016 May 12;14:132. doi: 10.1186/s12967-016-0894-9 (PMC4866013; doi:10.1186/s12967-016-0894-9)
Supplement: Supplementary file 1 — 10.1186/s12967-016-0894-9 The sequences of the primers used for real-time qPCR. [file 12967_2016_894_MOESM1_ESM.docx]

**Table S1. The sequences of the primers used for real-time qPCR.**

|  | Forward primer | Reverse primer |
| --- | --- | --- |
| IL-17A | 5’-ACCAATCCCAAAAGGTCCTC-3’ | 5’-GGGGACAGAGTTCATGTGGT-3’ |
| IFN-γ | 5’-GAGTGTGGAGACCATCAAGGA-3’ | 5’-CAGTTCAGCCATCACTTGGA-3’ |
| GAPDH | 5’-ATCATCAGCAATGCCTCC-3’ | 5’-CATCACGCCACAGTTTCC-3’ |
