# Supplementary figures and images for: Increased Th17 cells and IL-17A exist in patients with B cell acute lymphoblastic leukemia and promote proliferation and resistance to daunorubicin through activation of Akt signaling
Source: J Transl Med. 2016 May 12;14:132. doi: 10.1186/s12967-016-0894-9 (PMC4866013; doi:10.1186/s12967-016-0894-9)

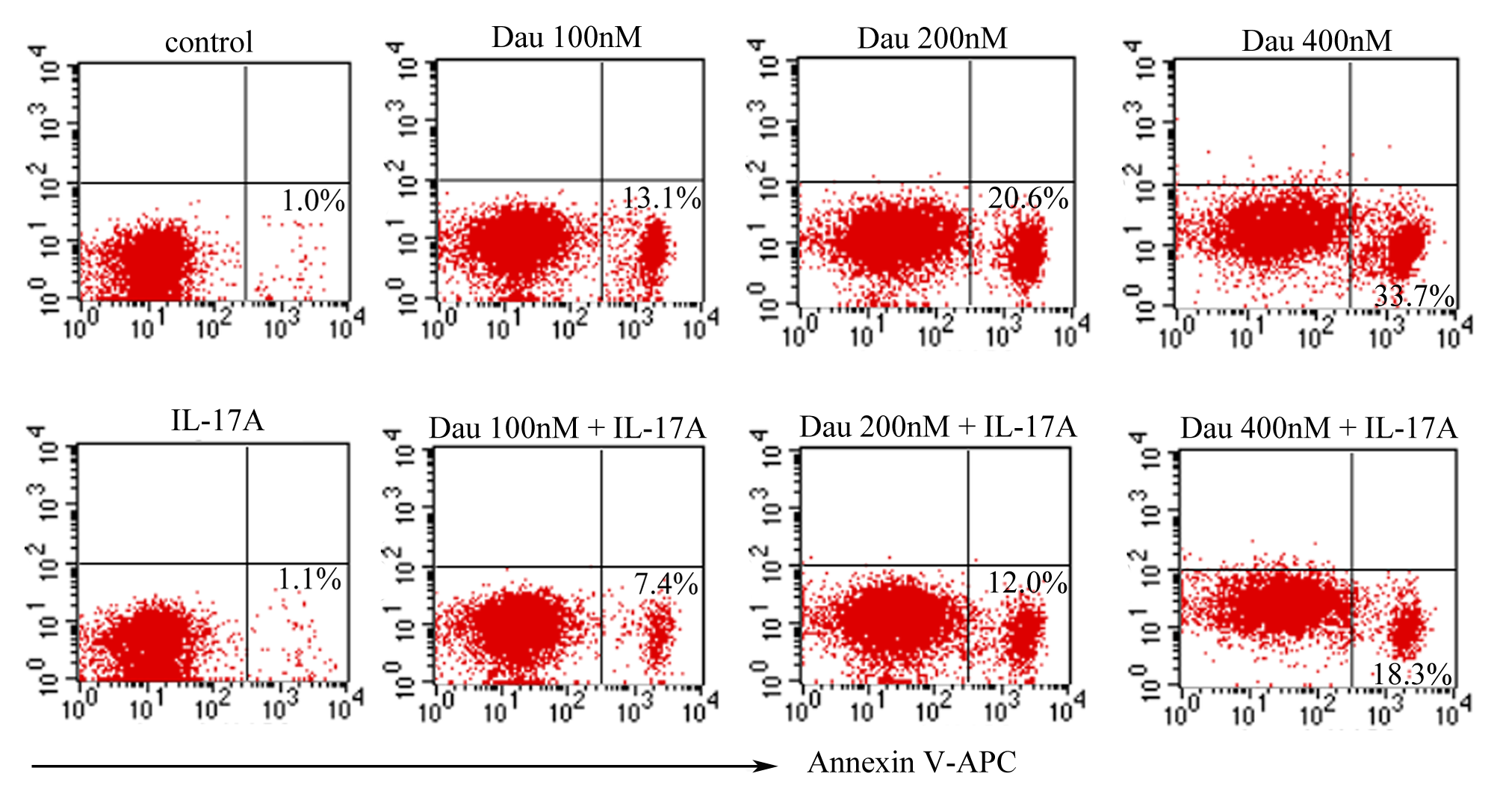

Supplement: Supplementary file 2 — 10.1186/s12967-016-0894-9 IL-17A reduces apoptosis to daunorubicin in Nalm-6 cells. Nalm-6 cells were cultured for 24 h in complete medium with or without IL-17A at 50 ng/ml and subsequently supplemented with daunorubicin at 100, 200, or 400 nM as indicated for 24 h. Apoptosis was determined using Annexin V staining on a flow cytometry. Images representing 3 independent experiments were shown. [file 12967_2016_894_MOESM2_ESM.tif]

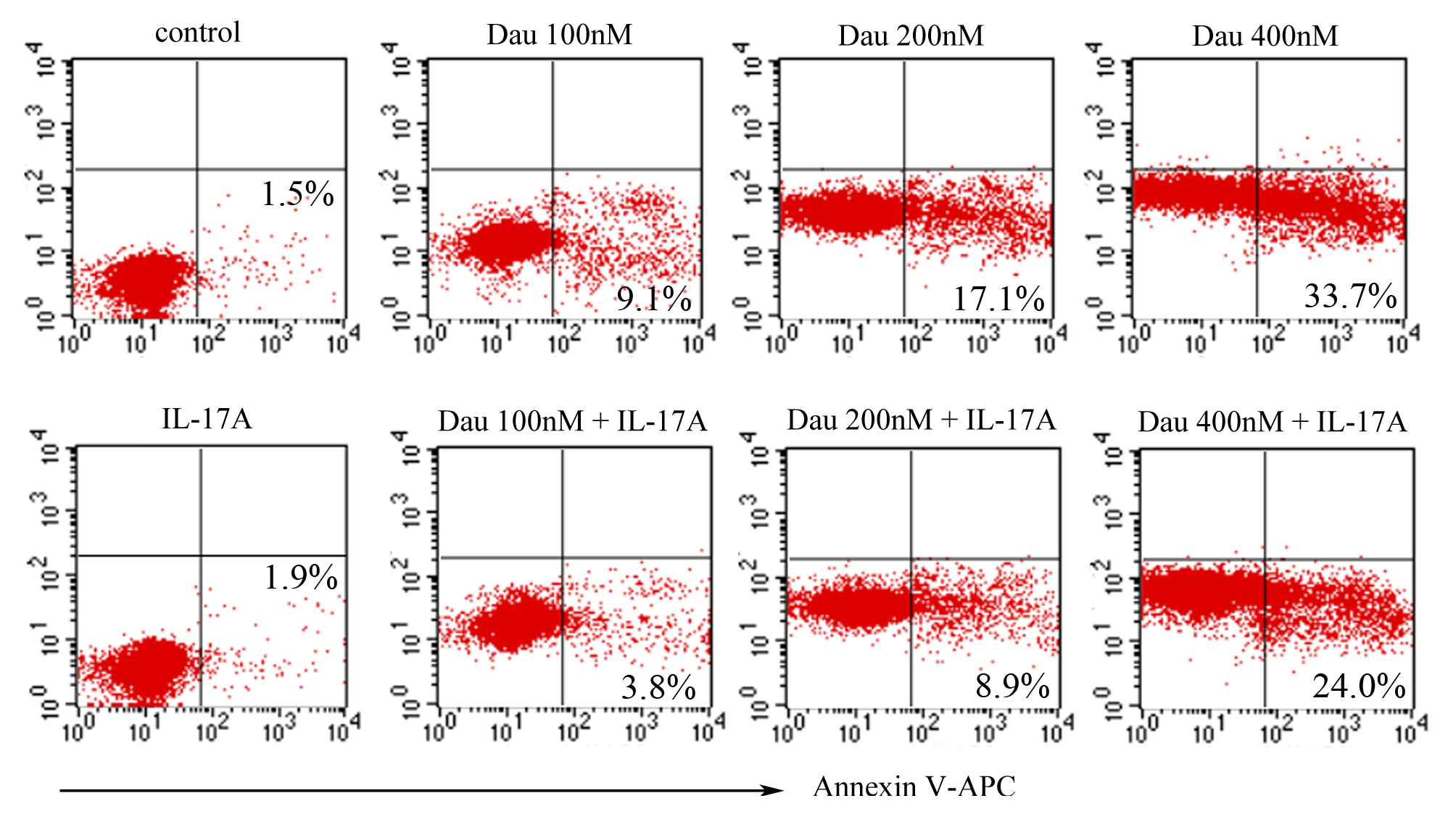

Supplement: Supplementary file 3 — 10.1186/s12967-016-0894-9 IL-17A reduces apoptosis to daunorubicin in patient B-ALL cells. Patient B-ALL cells isolated from 4 patients with B-ALL were cultured for 24 h in complete medium with or without IL-17A at 50 ng/ml and subsequently treated with daunorubicin at 100, 200, or 400 nM as indicated for 24 h. Apoptosis was measured using Annexin V staining on a flow cytometry. Images representing at least 3 independent experiments were shown. [file 12967_2016_894_MOESM3_ESM.tif]
